# Higher BMI is associated with reduced brain volume in heart failure

**DOI:** 10.1186/2052-9538-1-4

**Published:** 2014-02-19

**Authors:** Michael L Alosco, Adam M Brickman, Mary Beth Spitznagel, Atul Narkhede, Erica Y Griffith, Naftali Raz, Ronald Cohen, Lawrence H Sweet, Lisa H Colbert, Richard Josephson, Joel Hughes, Jim Rosneck, John Gunstad

**Affiliations:** Department of Psychology, Kent State University, Kent, OH USA; Taub Institute for Research on Alzheimer’s Disease and the Aging Brain, Department of Neurology, College of Physicians and Surgeons, Columbia University, New York, NY USA; Department of Psychiatry, Summa Health System, Akron City Hospital, Akron, OH USA; Institute of Gerontology, Wayne State University, Detroit, MI USA; Departments of Neurology Psychiatry and the Institute on Aging, Center for Cognitive Aging and Memory, University of Florida, Gainesville, FL 32611 USA; Department of Psychology, University of Georgia, Athens, GA USA; Department of Kinesiology, University of Wisconsin, Madison, WI USA; Department of Medicine, University Hospitals Case Medical Center, Cleveland, OH USA; Harrington Heart & Vascular Institute, Cleveland, OH USA; Case Western Reserve University School of Medicine, Cleveland, OH USA

**Keywords:** Brain volume, Cerebral blood flow, Heart failure, Neuroimaging, Obesity

## Abstract

**Background:**

Heart failure (HF) patients are at risk for structural brain changes due to cerebral hypoperfusion. Past work shows obesity is linked with reduced cerebral blood flow and associated with brain atrophy in healthy individuals, although its effects on the brain in HF are unclear. This study examined the association among body mass index (BMI), cerebral perfusion, and brain volume in HF patients.

**Results:**

Eighty HF patients underwent transcranial Doppler sonography to quantify cerebral blood flow velocity of the middle cerebral artery (CBF-V of the MCA) and brain magnetic resonance imaging (MRI) to quantify total brain, total and subcortical gray matter, white matter volume, and white matter hyperintensities. Body mass index (BMI) operationalized weight status. Nearly 45% of HF patients exhibited a BMI consistent with obesity. Regression analyses adjusting for medical variables, demographic characteristics, and CBF-V of the MCA, showed increased BMI was associated with reduced white matter volume (*p* < .05). BMI also interacted with cerebral perfusion to impact total gray matter volume, but this pattern did not emerge for any other MRI indices (*p* < 0.05).

**Conclusions:**

Our findings suggest increased BMI negatively affects brain volume in HF, and higher BMI interacts with cerebral perfusion to impact gray matter volume. The mechanisms for these findings remain unclear and likely involve multiple physiological processes. Prospective studies are needed to elucidate the exact pattern and rates of brain changes in obese HF persons.

## Background

Heart failure (HF) is associated with adverse medical outcomes, including greater risk of mortality and rehospitalization [1]. Past findings suggest that HF increases risk for neurological disorders such as Alzheimer’s disease and vascular dementia [2, 3]. However, structural brain differences can be observed in HF patients compared to age-matched controls prior to onset of these conditions. The wide-spread differences include smaller gray and white matter volumes, impaired axonal diffusion characteristics and increased white matter hyperintensities (WMH) [4–6].

Cerebral hypoperfusion and the resulting ischemia have been proposed to be the most significant contributors to adverse brain changes in patients with HF [5, 7–9]. Supporting such mechanisms is past work showing that reduced cerebral blood flow is prevalent and linked with neurocognitive consequences and structural brain damage in HF and other cardiovascular disease populations [10–15]. Consistent with this notion, the effects of common vascular risk factors (e.g., hypertension, diabetes, sleep apnea) on neurocognitive outcomes in HF are believed to stem from their negative impact on cerebral perfusion [16–19].

Obesity affects up to 40% of HF patients, and has adverse effects on brain volume in this population [20–22]. Indeed, obesity has been suggested to be an independent risk factor for structural brain changes. For example, among otherwise healthy older adults, increased BMI is associated with smaller whole brain and total gray matter volume [23], and obesity has been independently linked with smaller brain volume in patients with Alzheimer’s disease [24]. In addition to specific pathophysiological mechanisms, obesity may affect brain volume in HF through augmentation of other vascular risk factors via greater reductions in cerebral blood flow. For instance, obesity is associated with reduced cerebral perfusion in HF and the combination of these factors exacerbate cognitive impairment [20].

Despite these findings, no study to date has examined the association among body mass index (BMI), cerebral perfusion, and brain volume in persons with HF. The purpose of the current study was to examine the independent association between BMI and brain volume in older adults with HF. We then sought to determine whether elevated BMI and decreased cerebral perfusion interact to exacerbate brain volume reductions in this population.

## Methods

### Participants

This sample consisted of 80 persons with HF from an ongoing study of neurocognitive outcomes in HF. Strict inclusion/exclusion criteria were chosen for entry into the study. Specifically, the participants were between the ages of 50–85 years of age, native English speakers, and had an established diagnosis of New York Heart Association (NYHA) class II, III, or IV at the time of enrollment. Exclusion criteria included history of significant neurological disorder (e.g. dementia, stroke), head injury with more than 10-minutes loss of consciousness, severe psychiatric disorder (e.g. schizophrenia, bipolar disorder), history of substance use, and renal failure. Participants for the current study were also excluded for any contraindications to magnetic resonance imaging (MRI), such as cardiac pacemaker. Participants averaged 68.23 (SD = 8.58) years of age, and 35.0% of them were women. Medical record review revealed that the current sample exhibited an average left ventricular ejection fraction (LVEF) of 42.99 (SD = 13.32). See Table 1 for sample demographic and medical characteristics.

**Table 1:** **Demographic, medical, and cognitive characteristics of older adults with heart failure (** ***N*** **= 80)**

**Demographic characteristics**	
Age, mean (SD) years	68.23 (8.58)
Female (%)	35.0
Education, mean (SD) years	13.90 (2.76)
**Medical characteristics**	
LVEF %, mean (SD)	42.99 (13.32)
NYHA Class (% II, III, IV)	87.5, 11.3, 1.3
Hypertension (% yes)	70.0
Diabetes (% yes)	27.5
Sleep apnea (% yes)	22.5
Myocardial infarction (% yes)	57.5
Depression (% yes)	18.8
MCA CBF-V, mean (SD) cm/s	43.00 (13.56)
Body mass index, mean (SD) kg/m^2^	29.89 (6.68)

*NYHA* New York Heart Association; *LVEF* Left Ventricular Ejection Fraction; *MCA CBF-V* Cerebral Blood Velocity of the MCA.

### Measures

#### Neuroimaging

Whole-brain, high-resolution 3D T1-weighted images (Magnetization Prepared Rapid Gradient-Echo, MPRAGE) were acquired on a Siemens Symphony 1.5Tesla scanner for morphometry analysis. Twenty-six slices were acquired in the sagittal plane with a 230 × 100 mm field of view. The acquisition parameters were as follows: Echo time (TE) = 17, repetition time (TR) = 360, acquisition matrix = 256 × 100, and slice thickness = 5 mm. Whole-brain T2 and FLAIR images were also acquired to quantify WMH. For the T2-weighted images, twenty-one 5-mm thick slices were acquired with a 230 × 100 mm field of view with TR = 2910 and TE = 134. For the FLAIR images, twenty-one 5-mm slices were acquired with TR = 8500, TE = 115, and FOV = 220 × 75.

Morphometric analysis of brain structure was completed with FreeSurfer Version 5.1 (http://surfer.nmr.mgh.harvard.edu). Detailed methodology for regional and total volume derivation has been described in detail previously [25–27]. Briefly, FreeSurfer was used to preprocess images (e.g. intensity normalization, skull stripping) then provide an automated parcellation of cortical and subcortical structures via an automated processing stream. FreeSurfer performs parcellation by registering images to a probabilistic brain atlas, built from a manually labeled training set, and uses this probabilistic atlas to assign a neuroanatomical label to each voxel in an MRI volume. Total brain volume, total gray matter, subcortical gray matter, cortical white matter volume, and intracranial volume measurements are derived automatically.

#### Cerebral blood flow

Total white matter hyperintensities (WMH) volume was derived by a three-step operator-driven protocol that has been described in detail previously [28, 29]. Briefly, in Step 1, a threshold was applied to each FLAIR image to label all voxels that fell within the intensity distribution of hyperintense signal. In Step 2, gross regions-of-interest (ROI) were drawn manually to include WMH but to exclude other regions (e.g., dermal fat) that have similar intensity values. In Step 3, a new image is generated that contains the intersection of voxels labeled in Step 1 and those labeled in Step 2. The resulting image contains labeled voxels that are common in Step 1 and Step 2. The number of resulting voxels is summed and multiplied by voxel dimensions to derive a total volume score. We have shown the validity and reliability of this approach previously [28].

Transcranial Doppler ultrasonography through an expanded Stroke Prevention Trial in Sickle Cell Anemia (STOP) protocol [30] was used to assess mean cerebral blood flow velocity (CBF-V) of the Middle Cerebral Artery (MCA). The MCA irrigates the frontal, temporal, and the parietal cerebrum. It is sensitive to changes in cerebral blood flow and has also been suggested to be a more reliable representation of CBF-V relative to other TCD measured arteries (e.g., ACA, PCA) [31, 32]. In addition, relative to healthy controls persons with HF have a significantly lower CBF-V in the MCA [15].

#### Demographic and medical characteristics

Participant demographic and medical characteristics were ascertained through medical record review and self-report. See Table 1.

### Procedures

The Kent State University and Summa Health System Institutional Review Boards approved the study procedures and all participants provided written informed consent prior to study enrollment. All study procedures comply with the Declaration of Helsinki. For all participants, a medical chart review was performed and height and weight were measured. HF patients then underwent TCD and brain MRI.

### Statistical analyses

A square root transformation was applied to WMH to correct for a positively skewed distribution. Separate hierarchical regression models were used to examine the independent effects of BMI and CBF-V of the MCA on structural brain indices (e.g., total brain, total gray matter, subcortical gray matter, cortical white matter volume, and WMH volume). However, to limit the number of analyses and preserve power, for each volumetric index, one regression model that included both CBF-V of the MCA and BMI was performed to determine the effects of each of these variables on the MRI variables. Intracranial volume, as well as medical and demographic characteristics was entered in block 1. They included age, sex (1 = male; 0 = females), years of education, LVEF, diagnostic history of hypertension, diabetes, sleep apnea, myocardial infarction, and depression (1 = positive diagnostic history; 0 = negative diagnostic history). These medical and demographic variables were included as covariates in light of their known influence on neurocognitive outcomes in older adults with HF. CBF-V of the MCA was then entered as a block 2 variable and the continuous BMI variable was entered in block 3 to determine the incremental predictive validity of BMI and cerebral perfusion on brain volume in HF. Moderation analyses using hierarchical regression models were conducted to determine the synergistic effects of BMI and cerebral perfusion on the same volumetric indices listed above. Intracranial volume, BMI, and CBF-V of the MCA were transformed to z-scores and individually entered in block 1. The cross product of BMI and CBF-V of the MCA was computed and entered in block 2.

## Results

### Descriptive statistics

The sample mean BMI was 29.89 (SD = 6.68). By common categorization, 25.0% of the participants fell within the normal range (BMI = 18.5-24.9), 33.8% were overweight (BMI = 25 to 29.9) and 41.3% of the sample exhibited a BMI consistent with obesity (BMI ≥ 30). BMI groups did not differ in age, sex, education, LVEF, NYHA class, and frequency of diagnostic history of diabetes, myocardial infarction, and depression. In contrast, sleep apnea and hypertension were more common among the obese persons than among overweight and normal weight participants. See Table 2. Of note, bivariate correlations showed that higher BMI was associated with reduced CBF-V of the MCA (*r*(78) = −.22, *p* = .05).

**Table 2:** **Between BMI group differences among older adults with heart failure**

Demographic characteristics	Normal weight	Overweight	Obese	χ^2^/***F*** statistic
*N*	20	27	33	
Age, mean (SD) years	69.75 (8.80)	69.89 (8.44)	66.55 (8.52)	1.18
Sex (% Female)	40.0	25.9	39.4	.17
Years of education, mean (SD)	14.10 (3.01)	14.15 (3.11)	13.58 (2.32)	.38
**Medical characteristics**				
LVEF %, mean (SD)	40.15 (14.20)	43.00 (12.74)	44.70 (13.35)	.72
NYHA Class (% II, III, IV)	80.0, 20.0, 0.0	88.9, 7.4, 3.7	90.9, 9.1	3.99
Hypertension (% yes)	45.0	74.1	81.8	8.36**
Diabetes (% yes)	15.0	22.2	39.4	4.29
Sleep apnea (% yes)	10.0	14.8	36.4	6.34*
Myocardial infarction (% yes)	45.0	59.3	63.6	1.82
Depression (% yes)	20.0	14.8	21.2	.43

*Note. NYHA* New York Heart Association; *LVEF* Left Ventricular Ejection Fraction; *p < .05; **p < .01.

### The independent effects of BMI on brain volume

Models containing medical and demographic characteristics were associated with total brain volume, total gray matter volume, subcortical gray matter volume, cortical white matter volume, and WMH (*p* < .05 for all). See Table 3. After adjusting for medical and demographic variables, decreased CBF-V of the MCA was associated with increased WMH volume (β = −.25, *p* = .03). Significant findings for CBF-V of the MCA were not observed for any of the other brain volume indices (*p* > .05), although all values were in the expected positive direction (i.e., decreased CBF-V of the MCA and smaller brain volume).

**Table 3:** **BMI independently predicts structural brain volume in older adults with heart failure (** ***N*** **= 80)**

	WMH		TBV		Total GM		Subcortical GM		WM	
	***β***	SE b	***β***	SE b	***β***	SE b	***β***	SE b	***β***	SE b
*Block 1*										
Age, years	.23*	.02	-.11	1346.84	-.08	811.00	-.14	299.99	-.12	1799.30
Sex	-.15	.41	.01	32167.72	-.22	19369.89	-.12	7164.93	.12	42974.36
Education, years	-.29*	.05	.09	4304.00	-.01	2591.67	.16	958.66	.10	5749.92
LVEF %	-.15	.01	-.03	864.47	.03	520.54	-.03	192.55	-.04	1154.89
Hypertension	-.01	.34	.13	26933.65	-.18	16218.18	.04	5999.12	.20	35981.92
Diabetes	.08	.34	-.07	26560.24	-.11	15993.33	-.16	5915.94	-.05	35483.06
Sleep apnea	-.13	.37	.03	29212.06	.09	17590.13	.15	6506.60	.02	39025.75
MI	.11	.30	.05	23499.98	-.14	14150.58	-.16	5234.31	.08	31394.71
Depression	-.03	.39	.04	31194.14	-.07	18783.64	.01	6948.08	.06	41673.70
ICV	.23	.00	.81**	.08	.63	.05	.58**	.02	.53**	.11
*R* ^*2*^	.25		.67		.38		.35		.40	
*F*	2.25*		14.16*		4.19*		3.68**		4.65**	
*Block 2*										
CBF-V, cm/s	-.25*	.01	.08	918.63	.04	556.68	.09	205.01	.09	1229.93
*R* ^*2*^	.30		.68		.38		.36		.41	
*F* for ΔR^2^	6.67*		.98		.11		.71		.68	
*Block 3*										
BMI, kg/m^2^	.12	.03	-.16	2166.43	.19	1323.46	-.22	484.27	-.26*	2876.44
*R* ^*2*^	.30		.69		.40		.38		.45	
*F* for ΔR^2^	.78		3.35		2.22		3.12		4.53*	

*Note.* *p ≤ 0.05; **p < .01; sex: 1 = males and 0 = females; 1 = positive history and 0 = negative history for hypertension, diabetes, sleep apnea, MI, and depression.

Abbreviations: *β* – standardized regression coefficients, *SE* standard error; *BMI* Body Mass Index; *MI* Myocardial infarction; *ICV* Intracranial Volume; *CBF-V* Cerebral Blood Velocity of the MCA; *WMH* White Matter Hyperintensities; *GM* Gray Matter; *WM* White Matter Volume.

Volumetric indices units = mm^3^.

Hierarchical regression analyses controlling for medical and demographic characteristics and CBF-V of the MCA revealed that elevated BMI demonstrated a significant association with decreased white matter volume (β = −.26, *p* = .04) and strong trends for reduced subcortical gray matter volume (β = −.22, *p* = .08) and smaller total brain volume (β = −.17, *p* = .07). No such pattern emerged for total gray matter volume or WMH (*p* > .05 for each).

### Interactive effects between BMI and cerebral perfusion on brain volume

Moderation analyses using a hierarchical regression model showed a significant interaction between BMI and cerebral perfusion on total gray matter volume (β = .23, *p* = .03). However, no interactive effects between BMI and CBF-V of the MCA emerged for total brain volume (β = −.04, *p* = .59), subcortical gray matter volume (β = .14, *p* = .20), cortical white matter volume (β = −.10, *p* = .34), or WMH (β = .13, *p* = .31).

## Discussion

Consistent with past work, high BMI was prevalent and associated with reduced cerebral blood flow in this sample of older adults with HF. Obesity has recently been proposed as an independent risk factor for cognitive impairment in HF [20]. The current study extends these findings and shows that higher BMI adversely affects brain volume in this population and increased BMI exacerbated the effects of cerebral hypoperfusion on reduced gray matter volume. Several aspects of these findings warrant brief discussion.

The current study suggests that elevated BMI is independently associated with reduced structural brain volume in older adults with HF. There are several possible mechanisms for such findings. First, obesity promotes vascular risk factors (e.g., hypertension, diabetes) that are known to produce structural brain changes, even in healthy adults [33, 34]. In contrast, our findings and other work in otherwise healthy samples suggest obesity and the accompanying presence of adiposity may introduce unique pathophysiological mechanisms to produce brain changes in HF [23]. For instance, obesity is associated with altered levels of circulating biomarkers, including leptin [35], ghrelin [36], and brain derived neurotrophic factor (BDNF) [37], among others. These biomarkers are important for metabolism regulation and body weight and also promote neuronal survival, neurogenesis, dendritic synaptic formations, and reducing apoptosis of neurons—all biological processes that shape the cerebral structure [38–40]. In addition, obesity affects the brain via promotion of inflammatory processes, with a substantial contribution of genetic variants such as Fat Mass and Obesity (*FTO)* gene [41]. Future work is needed to clarify the exact mechanisms between BMI and brain volume in HF, particularly as they relate to the above physiological processes, especially inflammation that is a core feature of obesity and the associated metabolic syndrome [42].

The current study suggests that decreased cerebral perfusion is associated with increased WMH in persons with HF, but not with the other MRI indices. Cerebral hypoperfusion and subsequent development of WMH is the widely theorized mechanism of cognitive impairment in HF [7, 9]. Interestingly, higher BMI interacted with cerebral perfusion to impact gray matter volume, but not WMH. The exact reason for this pattern of findings is not entirely clear. A likely explanation may involve a threshold effect between obesity, cerebral perfusion, and WMH. For instance, given the prevalence of white matter damage in HF persons and its close association with brain hypoperfusion, it is possible that the additive effects of obesity may not be significant enough to modify this relationship. Moreover, WMH commonly precede brain atrophy [43] and it is possible that obesity accelerates this conversion. Nonetheless, the relationship between obesity and neurocognitive outcomes in HF may be more complicated than believed and involve other mechanisms beyond cerebral hemodynamics (e.g., altered adipokine levels, inflammation, genetic contributors). Future work is much needed to elucidate the effects of high BMI and cerebral hypoperfusion on the brain in HF patients.

The current study is limited in several ways. First, the current study consisted of cross-sectional analyses and case-controlled prospective studies are needed to determine whether higher BMI accelerates brain atrophy in HF. In addition, although BMI is practical and widely used it remains a coarse measure of obesity. More precise and detailed assessments of obesity (e.g., dual-energy x-ray absorptiometry) would provide key insight into the effects of obesity on the brain in HF through its ability to distinguish between bone, fat, and lean tissue. DEXA imaging would also help clarify the regional effects of obesity on cerebral morphometry (e.g., abdominal vs. non-abdominal obesity). Similarly, although TCD is a non-invasive and reliable measure of cerebral blood flow [32], it is a non-direct assessment of cerebral perfusion and future studies should employ arterial spin labeling, phase contrast MRI, or positron emission tomography to elucidate the interaction between cerebral perfusion, obesity, and brain volume in HF. The current study also did not employ a control group and thus we attempted to statistically control for many medical and clinical variables that are known to influence neurocognitive outcomes in HF. As a result, the power of analyses was reduced and larger studies that utilize healthy controls are much needed to confirm the current findings. We also tested the moderating effects of cerebral perfusion on the association between BMI and brain volume, and studies with larger samples should use model-based approaches to determine the mediating properties of perfusion in this relationship. Lastly, the effect sizes for the impact of BMI on brain volume were modest and prospective studies with larger samples would help clarify the clinical meaningfulness of the current findings.

## Conclusion

In summary, the current study shows that higher BMI is an independent contributor to reduced brain volume in older adults with HF. The mechanisms of this relationship may involve altered cerebral hemodynamics, but are likely complex and involve multiple physiological processes. Prospective studies are needed to confirm the effects of obesity on neuroimaging indices and clarify the etiological underpinnings.
